# Tp40: a new potential prognostic and diagnostic marker for syphilis

**DOI:** 10.1128/spectrum.02799-24

**Published:** 2025-02-11

**Authors:** Jiangchen Yao, Bibo Xie, Xuan Ding, Han Yu, Ting Lin, Junxia Duan, Xiaohong Zhang, Peng Ling, Feijun Zhao

**Affiliations:** 1 MOE Key Lab of Rare Pediatric Diseases&Institute of Pathogenic Biology and Key Laboratory of Special Pathogen Prevention and Control of Hunan Province, Hengyang Medical College, University of South China, Hengyang, China; 2 Department of Clinical Laboratory Medicine, The Central Hospital of Shaoyang, Shaoyang City, China; 3 Department of Critical Care Medicine, The Central Hospital of Shaoyang, Shaoyang City, China; 4 Department of Clinical Laboratory Medicine, The First Affiliated Hospital, Hengyang Medical College, University of South China, Hengyang, China; 5 Department of Clinical Laboratory Medicine, Changsha Central Hospital, Changsha, China; Southern Medical University, Guangzhou, China

**Keywords:** *Treponema pallidum*, Tp40, infection phase-dependent antigen, serodiagnosis, protein characterization

## Abstract

The characterization of *Treponema pallidum* proteins is of great significance for the study of the prevention, diagnosis, and pathogenesis of syphilis. The structures and functions of many *T. pallidum* proteins, including the Tp40 (Tp0134) protein, remain unknown. To explore the expression pattern of the Tp40 protein within *T. pallidum*, we established an animal model of syphilis infection to compare the variations in serum Tp40 antibody levels between Live and Inactivated Tp groups. The results indicated that the absorbance of Tp40-enzyme-linked immunosorbent assay (ELISA) did not increase in the Inactivated group and the Untreated group, but it increased in the Live Tp infection group, suggesting that the Tp40 protein is an *in vivo*-induced antigen that is only actively expressed during infection. In addition, the localization of the Tp40 protein was determined by the gel microdrop method. We found that Tp40 may be a transmembrane protein with a signaling peptide present in the intima periplasm of *T. pallidum*. Finally, 468 patients’ sera were collected for diagnostic value evaluation. Tp40-ELISA, LZ-ELISA, and Shanghai Kehua rapid plasma reagin (RPR) reagent kit showed a high degree of consistency in 468 serum samples. This suggests that Tp40 could be a valuable diagnostic antigen. The results of this study provide a new reference for the study of the pathogenesis, protein function, and diagnosis of syphilis.

**IMPORTANCE:**

In recent years, syphilis, as a chronic infectious disease, has once again attracted much attention. *Treponema pallidum* exhibits remarkable infectivity, concealment, and aggressiveness, posing considerable challenges to its prevention and control. The underlying pathogenic mechanisms remain elusive, and during the infection process, the roles of numerous proteins are still unclear. Through protein characterization in this study, it was found that the Tp40 protein is highly likely to be a transmembrane protein with a signal peptide and may be located in the periplasm. Besides, based on experiments with animal models and the detection of human serum samples, we believe that the Tp40 protein is a potential *in vivo*-induced antigen of *T. pallidum* that can be used for serological diagnosis of syphilis. This study conducted a preliminary exploration of the Tp40 protein and provided a meaningful reference for further exploration of the functional mechanism of the Tp40 protein and its significance in clinical diagnosis.

## INTRODUCTION

Syphilis is a sexually transmitted infection caused by the bacterium *Treponema pallidum (Treponema pallidum* subsp. *pallidum*), which can lead to multiple stages of chronic and multisystemic disease. Its persistence and ability to affect various systems in the body make it a serious and potentially debilitating condition if left untreated (1). According to the data from the Chinese Center for Disease Control and Prevention (http://www.chinacdc.cn), in China, infectious diseases are classified into three categories: Category A, Category B, and Category C. Category A infectious diseases refer to two extremely infectious and fatal diseases with high mortality rates that require mandatory management, namely plague and cholera. Category B infectious diseases include many diseases such as severe acute respiratory syndrome, acquired immunodeficiency syndrome, and viral hepatitis. The harmfulness of these diseases is relatively lower than that of Category A diseases, but they also need to be managed and prevented in strict accordance with the prevention and control requirements. Syphilis belongs to Category B infectious diseases. In the past 15 years, it ranked third among Category B infectious diseases in China and is a major sexually transmitted disease. In 2016, the World Health Organization established the “Global Health Sector Strategy on Sexually Transmitted Infections,” with the goal of reducing the incidence of syphilis by 90% by 2030 globally and reducing the incidence of congenital syphilis to below 0.05% before 2030 in 80% of countries (2).

To tackle this challenge, researchers have adopted three approaches: studying the pathogenic mechanisms of *T. pallidum*, searching for highly specific and sensitive diagnostic markers, and developing effective syphilis vaccines. Currently, although significant progress has been made in the *in vitro* coculture of *T. Pallidum* (3, 4), achieving large-scale, low-cost artificial cultivation is still not mature (5). There are various laboratory diagnostic methods for syphilis, but their practicality is limited by the nature of the disease and the inherent limitations of the methods. Serological testing has been the mainstay of syphilis laboratory diagnosis, but there are still significant challenges in the serological diagnosis of congenital syphilis and neurosyphilis, including the lack of specificity in non-treponemal tests and the poor correlation between treponemal tests and disease activity (6, 7). Direct detection methods, such as molecular or specialized microscopy techniques, also have limitations in terms of performance, availability, and cost. The most widely used serological diagnostic method for syphilis is the combination of *Treponema pallidum* particle agglutination test (TPPA) with rapid plasma reagin test (RPR) or toluidine red unheated serum test (TRUST) to determine disease activity (7). The unique outer membrane characteristics of *T. pallidum* result in limited exposure to surface antigens, and the surface antigen gene sequences of *T. pallidum* can exhibit diversity, such as TprK. This may be an important reason for the poor efficacy of syphilis vaccines and immune escape of *T. pallidum*, considering the phenomenon of “original antigenic sin” observed in antibody responses to influenza A virus infection or vaccination (4, 8). Continued research on the identification and function of unknown hypothetical proteins of *T. pallidum* is crucial for a deeper understanding of its pathogenic mechanisms, the search for diagnostic markers, and the development of syphilis vaccines.

Research has shown that inducible genes in the host can be a determining factor in the virulence of certain pathogens; they play an important role in pathogen infection and pathogenesis (9). The emergence of *in vivo*-induced antigen technology (10) has greatly facilitated the discovery, prevention, diagnosis, drug development, and pathogenic mechanisms related to *in vivo*-induced antigens of pathogens such as *Vibrio cholerae*, *Chlamydia psittaci*, and *Mycoplasma pneumoniae* (11–13). Similarly, in the active stage of *T. pallidum* infection, *in vivo*-induced antigens may also be produced (14). During the previous bioinformatics analysis of the *T. pallidum* whole genome, our research group found that the hypothetical protein Tp0134 (Tp40) of *T. pallidum* may possess the characteristics of an *in vivo*-induced antigen (14, 15). However, there are still many unknowns in the study of the Tp40 protein. It is only known to belong to the same family as Tp0136 and Tp0462 (16). Moreover, the description of this protein in the protein database Uniprot is not comprehensive (https://www.uniprot.org/uniprotkb/O83170/entry). Therefore, this study conducts relevant experimental verification on this novel *T. pallidum* hypothetical protein based on protein signaling peptides and transmembrane domain prediction for the first time, in order to preliminarily identify and explore its potential clinical application value.

## MATERIALS AND METHODS

### Materials

#### Experimental animals

New Zealand white rabbits (SYXK Xiang 2020-0002) were provided and housed by the Experimental Animal Department of Hengyang Medical College, University of South China. The rabbit housing room was maintained at a temperature of 18°C–20°C, and they were fed with antibiotic-free feed and water.

#### Strain

*T. pallidum* Nichols standard strain (14) was generously provided by Professor Yang Tianci (Director of the Clinical Laboratory Center, Xiamen University Affiliated Zhongshan Hospital, China). As described above (17) adult male New Zealand white rabbits were inoculated and passaged.

#### Sample sources

A total of 468 patients were included, consisting of 264 males (56.4%) aged 0–88 years and 204 females (43.6%) aged 0–76 years. Serum samples from syphilis patients and healthy individuals were collected from the First Affiliated Hospital of the University of South China, the Second Affiliated Hospital of the University of South China, and the First Hospital of Changde City, Hunan Province. Syphilis patients were diagnosed according to industry standards (Guidelines for diagnosis and treatment of syphilis, gonorrhea, and genital Chlamydia trachomatis infection [2020]), confirmed by clinicians based on patients’ clinical manifestations, laboratory tests, and medical histories. After excluding patients with coexisting diseases (such as HIV, systemic lupus erythematosus, etc.), pregnant women, individuals with allergies, and those who declined to participate, a total of 214 syphilis patients were enrolled. To evaluate the diagnostic specificity of the Tp40-enzyme-linked immunosorbent assay (ELISA), we conducted an assessment using serum samples from healthy individuals and individuals with diseases potentially causing false-positive syphilis results. Specifically, we collected a total of 194 serum samples from healthy individuals, 30 serum samples from Lyme disease patients sourced from the Chinese Center for Disease Control and Prevention, and 30 serum samples from leptospirosis patients obtained from the Hunan Provincial Center for Disease Control and Prevention. All participants provided informed consent.

### Methods

#### Protein bioinformatics analysis websites

##### Protein signal peptide prediction website

The SignalP online tool (https://services.healthtech.dtu.dk/service.php?SignalP) was used to determine the presence of signal peptides in the sequences. The protein may have a Sec signal peptide (Sec/SPI), lipoprotein signal peptide (Sec/SPII), Tat signal peptide (Tat/SPI), Tat lipoprotein signal peptide (Tat/SPII), Pilin signal peptide (Sec/SPIII), or no signal peptide (other) (18).

##### Protein transmembrane domain prediction website

The TMHMM2.0 online server (https://services.healthtech.dtu.dk/service.php?TMHMM-2.0) was used to predict potential transmembrane domains.

### Cloning, expression, purification, and identification of recombinant protein Tp40

The Tp40 gene was amplified from *T. pallidum* Nichols strain genomic DNA using polymerase chain reaction (PCR). The forward primer 5′-CGCGGATCCTCACTCGCGATTCCAGGT-3′ (BamH I) and reverse primer 5′-CCGCTCGAGATGTGCAAACCGCGCGTGTGGCG-3′ (Xho I) were used. The amplified gene was cloned into the prokaryotic expression vector pET28a (Merck Millipore, Germany) to construct the recombinant plasmid. Positive clones were selected and sent for sequencing identification by Shanghai Biological Engineering Company. The plasmids were then transformed into expression strain *Escherichia coli BL21* (DE3). The Tp40 recombinant protein (rTp40) was expressed in large quantities under the conditions of 30°C and 0.3 mmol/L isopropyl β-D-1-thiogalactopyranoside induction. The ultra-wide range Western exposure protein Marker (Mei5bio, China) was used to identify rTp40 with western blot, and the chemiluminescence imaging system (Syngene, UK) was used to preserve the images. The endotoxin was removed using a polymyxin B column (GenScript, USA), and the protein concentration was determined using the BCA assay kit (Beyotime Biotechnology, China).

### Preparation and quantification of virulence *T. pallidum*

This strain was propagated with the rabbit infectivity test and resuspended to obtain Live *T. pallidum* suspension referred to the previous studies. To obtain an Inactivated *T. pallidum* suspension, the organisms were inactivated by heating at 56°C for 60 min. The number of *T. pallidum* was determined using the dark field microscopy enumeration method. Placed 10 µL of *T. pallidum* suspension on a microscope slide and covered with a 22 × 22-mm coverslip. Counted the number of *T. pallidum* in 100 microscope fields, being certain to count *T. pallidum* in all planes of focus in each field. Calculated the number of *T. pallidum* per mL of suspension: *T. pallidum*/mL = (total treponemes counted in 100 fields)/(100 fields × field vol in mL).

Standard curve establishment: the bacterial concentration of the suspensions was serially diluted in 10-fold increments from 10⁷/mL to 10²/mL. The logarithm base 10 of the concentrations of the *T. pallidum* suspensions after 10-fold serial dilutions were plotted on the x-axis and the Ct values obtained from real-time quantitative polymerase chain reaction (RT-qPCR) were plotted on the y-axis to draw the standard curves.

### Agarose gel coating

Indirect immunofluorescence assay: the experimental technique was based on the complete publication of Luthra et al. (19). In the experiment described in “The BAM Complex,” the following techniques were used: *T. pallidum* was encapsulated in agarose gel (Sigma-Aldrich, USA) to protect the fragile outer membrane of *T. pallidum*. The group of *T. pallidum* with an intact outer membrane, the group of *T. pallidum* whose membrane had been permeated by Triton X-100, and the group of *T. pallidum* lacking an outer membrane were labeled as intact, permeabilize, and removed, respectively. In the removed group, *T. pallidum* was collected via centrifugation for 20 min at 10,000 × *g* at 4°C. The precipitates were resuspended in ice-cold 0.05 M sodium citrate buffer and incubated on a rocker every 20 min vortexing at 25°C three times. The samples were centrifuged at 12,000 × *g* for 30 min, and the precipitate was washed twice in sterile 0.9% saline. *T. pallidum* samples were treated with 0.05 M citrate buffer, which dissociates endoflagellar filaments and results in the complete release of the outer membrane *T. pallidum* treated with sodium citrate was encapsulated in gel microdroplets. In the intact, the treponeme suspension was heated at 56°C for 1 h to inactivate the organisms. The encapsulated *T. pallidum* was then transferred to 500 µL of normal saline containing 10% bovine serum. In the permeabilized group, a normal saline solution containing 0.2% Triton X-100 and 10% bovine serum was used for permeabilizing the outer membrane. The interaction of detergents with biological membranes includes the insertion of the hydrophobic portions of detergents into the hydrocarbon-like interior of the lipid phase and the dissolution of lipids on the membrane. Three groups were incubated at 4°C overnight (20).

Mouse anti-rTp0136, mouse anti-rTp40, and mouse anti-Tp0664 (*T. pallidum* periplasmic flagellar sheath protein, FlaA2) antibodies were prepared by immunizing BALB/c mice with recombinant protein, diluted 1:100 and added to the encapsulated *T. pallidum*. The mixture was incubated overnight at 4°C and washed five times with physiological saline. Then, a mixture of 400 µL of Alexa Fluor 488-labeled anti-mouse IgG secondary antibody (Abcam, UK) diluted 1:2,000 and DAPI stain (Solarbio, China) diluted 1:1,000 was added to the samples. The samples were incubated for 8 h at 4°C, washed five times, and carefully transferred to glass slides. The samples were observed using a fluorescence-inverted microscope (Nikon, Japan).

### Monitoring the transcription level of Tp40

Fresh New Zealand rabbit skin lesions infected with *T. pallidum* were obtained. Briefly, after intradermal inoculation of 10^6^ *T. pallidum* bacteria in each site on the back skin of four rabbits, the hair was removed daily. On days 4, 7, 11, 14, 18, 21, 25, and 28 after infection, Live lesion tissues from each inoculation site were collected. The lesion tissues were aseptically cut with a surgical blade, and RNA was extracted from the tissues using Trizol reagent (TIANGEN, China). The extracted RNA was reverse transcribed using a reverse transcription kit (KR118 TIANGEN, China). An RT-qPCR protocol, established in the laboratory, was used for relative quantification with an external standard. The mRNA levels of the target genes in *T. pallidum* were normalized to the mRNA level of the Tp0574 gene (encoding Tp47 lipoprotein) in the same sample (21, 22).

### Animal serological ELISA

As described previously (14), 15 New Zealand rabbits were randomly divided into the Live Tp group (six rabbits), Inactivated Tp group (six rabbits), and Untreated group (three rabbits) to establish a New Zealand rabbit infection model. In the Live Tp group, 1 mL of *T. pallidum* suspension with a concentration of approximately 1 × 10^7^ was injected intradermally on the back. In the Inactivated Tp group, the Inactivated Tp group was inactivated in a 56°C water bath for 1 h before intradermal injection. The blank group was inoculated with sterile phosphate-buffered saline (PBS).

Blood serum was collected and separated from the rabbits at weekly intervals within 8 weeks after infection, with an additional interval of 30–40 days. Serological tests for syphilis, including Treponema pallidum particle agglutination assay (TPPA, FUJIREBIO, Japan) and Rapid plasma reagin test (RPR, Kehua Bioengineering, China), were performed. ELISA plates (Costar, USA) were coated with purified recombinant protein rTp40 at a concentration of 1 mg/L. The outer membrane protein Tp92 was used as a control. An indirect immune ELISA method was used to detect changes in Tp40 protein antibodies. The serum samples were used as the primary antibody (diluted 1:200), and horseradish peroxidase-conjugated goat anti-human/rabbit IgG (Invitrogen, USA) was used as the secondary antibody (diluted 1:10,000). The Multiskan MK3 automated ELISA reader was used to measure the absorbance (A_450 nm_ OD value) at a wavelength of 450 nm for each well. Each sample was measured three times.

### Clinical serological ELISA

As described above, an indirect immune ELISA method based on the Tp40 protein (Tp40-ELISA) was used to detect specific anti-Tp40 antibodies in 468 clinical serum samples, including syphilis-positive serum, normal human serum, and cross-reactive serum. In addition, the 468 serum samples were tested using the TPPA assay, RPR test, and Zhuhai Lijiu syphilis screening ELISA kit (Lijiu Reagents, China). All samples were tested three times, and the average values were calculated.

### Statistical analysis

The experimental data were analyzed using SPSS software (version: 23.0). The diagnostic test comparison was conducted using a fourfold table method. The serological identification of induced antigens in animals was analyzed using repeated measures analysis and one-way analysis of variance (ANOVA). The Tp40-ELISA A_450 nm_ OD values were considered continuous data, and the changes in RPR titers were considered ordinal data, analyzed using Spearman’s rank correlation analysis. A significance level of *P* < 0.05 was considered statistically significant.

## RESULTS

### Bioinformatics prediction suggests that Tp40 is likely a non-transmembrane protein with a signal peptide

The SignalP 6.0 server can predict the presence of signal peptides and their cleavage sites in proteins from archaea, Gram-positive bacteria, Gram-negative bacteria, and eukaryotes. The amino acid sequence of the Tp40 (Tp0134 gene) protein was downloaded from the predicted coding region Tp0134 of *Treponema pallidum* subsp. *pallidum strain Nichols* (GenBank: AAC65125.1). The amino acid sequence of Tp40 in FASTA format was inputted into the search box. The prediction from SignalP 6.0 suggests that Tp40 has a lipoprotein signal peptide (Sec/SPII) with a probability of 0.954141. The predicted cleavage site of the signal peptide is located between the 29th and 30th amino acids, and the bioinformatics analysis diagram is appended in the supplementary materials.

Furthermore, we used TMHMM-2.0 to predict the presence of transmembrane helices in Tp40. After analysis, Tp40 has zero predicted transmembrane helices. Additionally, the expected number of amino acids in transmembrane helices is 7.61193, which is less than 18. Generally, the formation of a transmembrane helical structure necessitates approximately 18 to 20 amino acids, allowing it to maintain sufficient length to traverse the hydrophobic core of the membrane and sustain a stable structure. This suggests that Tp40 is unlikely to be a transmembrane protein. The expected number of amino acids in transmembrane helices within the first 60 amino acids of Tp40 is 7.60443, indicating that the N-terminus of Tp40 may be a signal peptide, consistent with the previous signal peptide prediction. However, the overall probability of the N-terminus of Tp40 being located on the cytoplasmic side of the cell membrane is only 0.37339, suggesting that Tp40 may not be a transmembrane protein. The graphical representation of the transmembrane domain analysis of Tp40 is shown in supplementary materials.

### Induction and identification of recombinant protein Tp40

**Fig 1:**
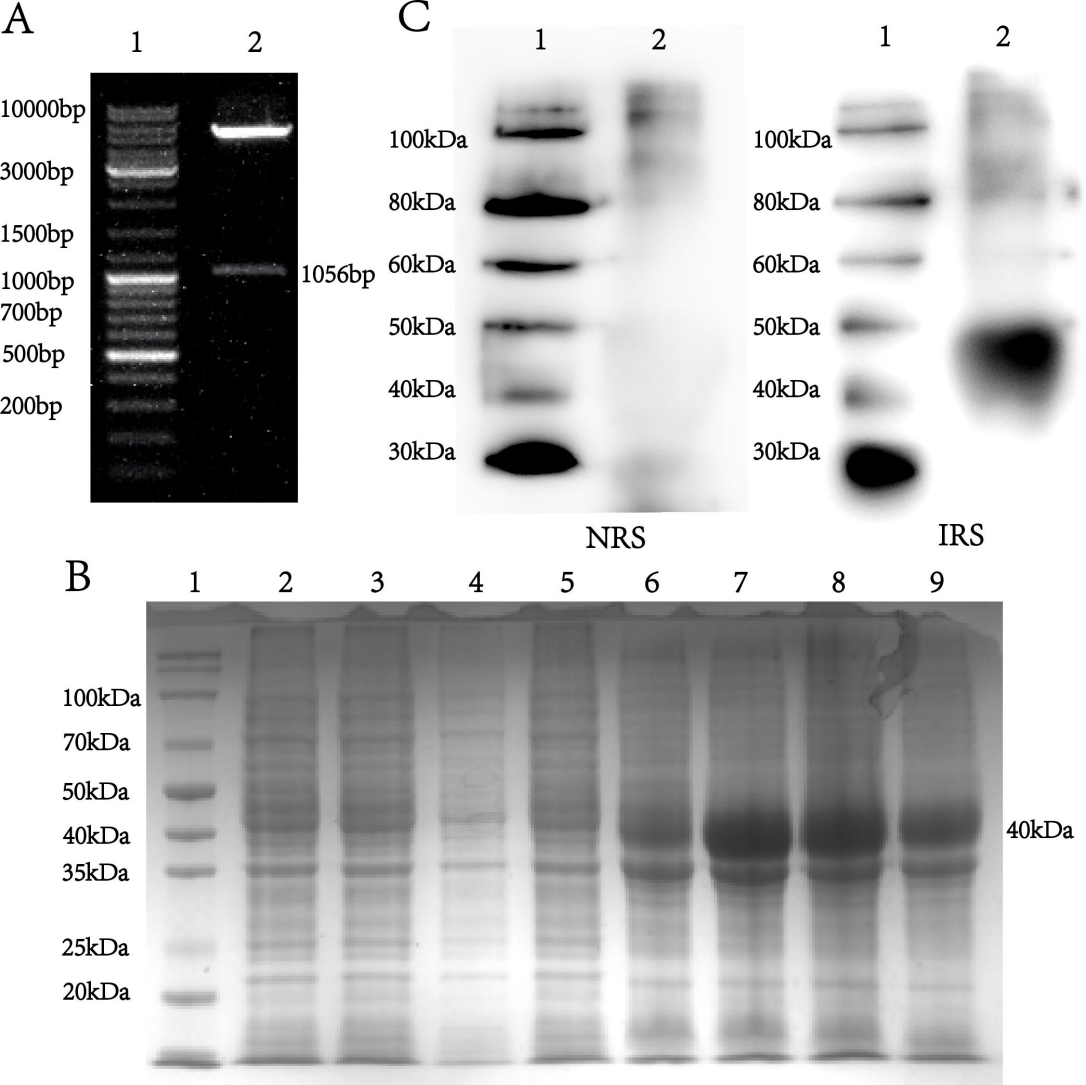
The double enzymatic digestion of recombinant plasmid DNA of Tp40 and the expression of Tp40 protein. (**A**) The restriction endonuclease map of recombinant plasmid pET28a (+)-Tp40 treated with BamH I and Xho I. The expression (**B**) and expression western blot (**C**) of pET28a (+)-Tp40 in *E.coli BL21* (DE3), and the Identified protein band, about the 40 kDa, is consistent with the molecular weight of the target protein. M: DNA marker; 1: marker of protein; 2: immunoblotting band; NRS, negative rabbit serum; IRS, infected rabbit serum.

The Tp40 (Tp0134) gene sequence was downloaded from the *Treponema pallidum* subsp. *pallidum str. Nichols* complete genome (GenBank: AE000520.1). Successful construction of the positive clone was confirmed by double enzyme digestion, as shown in Fig. 1A. A band of approximately 1,000 bp was observed, consistent with the expected size, and sequencing confirmed that the inserted gene sequence in the recombinant plasmid matched the target sequence. The recombinant protein Tp40 was successfully induced and purified, as shown in Fig. 1B. A protein band of approximately 40 kDa was observed on SDS-PAGE gel, and the purity was above 90%. Using rabbit syphilis-positive and negative serum as the primary antibody and goat anti-rabbit antibody as the secondary antibody, the protein was identified as the target protein through protein immunoblotting (Fig. 1C).

### The membrane localization detection of Tp40 suggests its presence in the periplasmic space between the inner and outer membranes of *T. pallidum*

**Fig 2:**
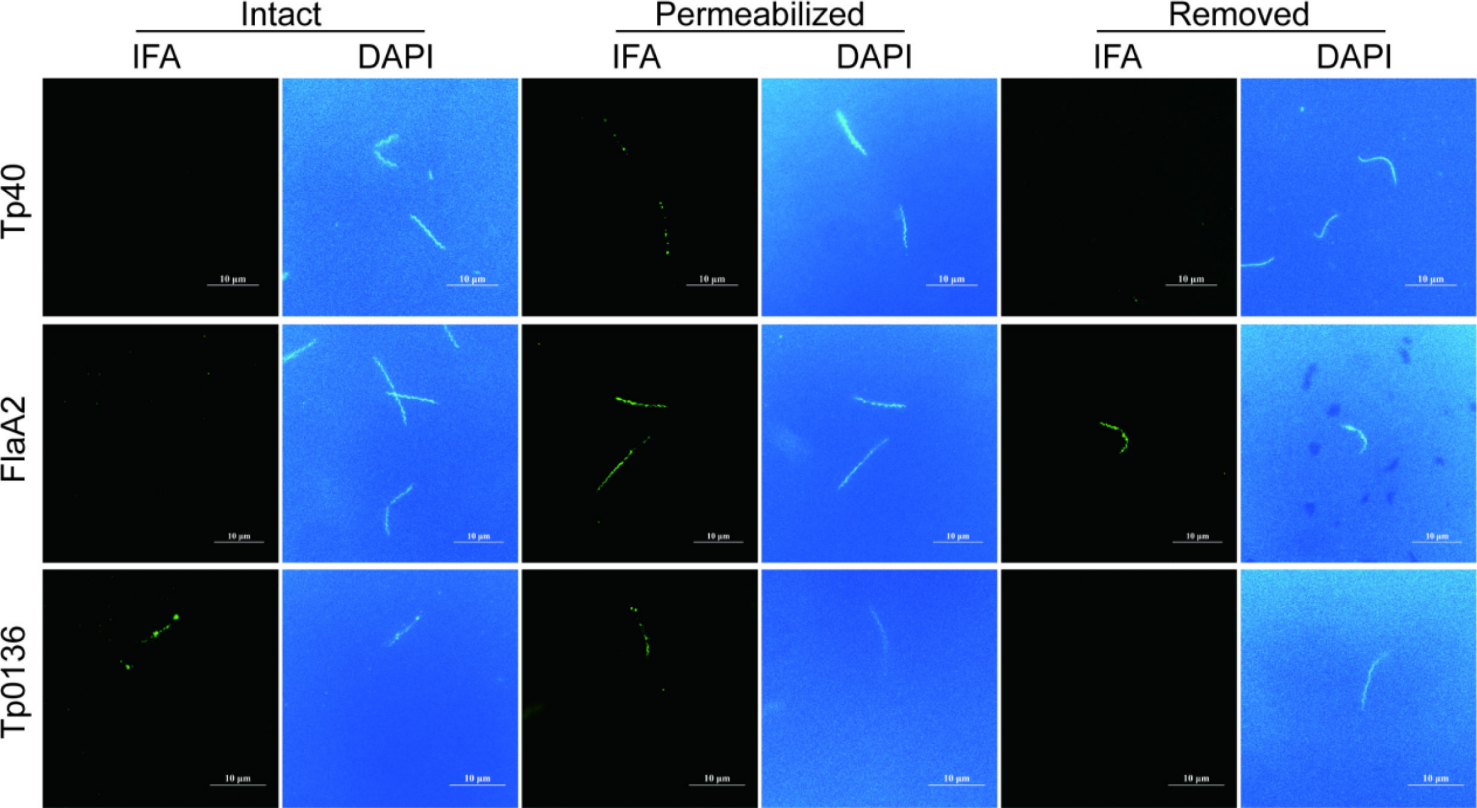
Membrane topology assessment of Tp40 in *T. pallidum*. *T. pallidum* bacteria encapsulated in agarose gel were exposed to a mouse antibodies anti-Tp40, anti-FlaA2(Tp0664), and anti-Tp0136 in the presence or absence of 0.2% (vol/vol) Triton X-100 (TX) or removed the outer membrane, separately. After secondary antibody exposure (anti-mouse Alexa Fluor 488), spirochetes were examined by fluorescent microscopy.

To verify whether Tp40 is a membrane protein of *T. pallidum*, pre-prepared mouse-derived recombinant protein antibodies were used to incubate *T. pallidum* intact outer membrane fractions, Triton X-100 permeabilized fractions, and *T. pallidum* membrane-depleted fractions. The results of each experimental group were observed using fluorescence microscopy, as shown in Fig. 2. The background staining with DAPI appeared as a blue color, indicating the dispersion of the dye in low-melting agarose. After DAPI staining, *T. pallidum* exhibited bright blue fluorescence, indicating successful labeling of *T. pallidum*.

In the control experiment, *T. pallidum* intact outer membrane fractions incubated with mouse-derived FlaA-2 antibodies did not show protein immunofluorescence. However, both Triton X-100 permeabilized fractions and *T. pallidum* membrane-depleted fractions showed bright blue fluorescence, consistent with the fact that FlaA-2 is located in the periplasmic space on the outer side of the inner membrane of *T. pallidum* (23). Similarly, *T. pallidum* intact outer membrane fractions and Triton X-100 permeabilized fractions incubated with mouse-derived rTp0136 antibodies exhibited bright blue fluorescence, but *T. pallidum* membrane-depleted fractions did not show green fluorescence, confirming once again that Tp0136 is an outer membrane protein of *T. pallidum* (16). When *T. pallidum* intact outer membrane fractions and *T. pallidum* membrane-depleted fractions were incubated with mouse-derived rTp40 antibodies, no protein immunofluorescence was observed. However, the Triton X-100 permeabilized fractions showed fluorescence, suggesting that Tp40 may be located in the periplasmic space on the outer side of the inner membrane of *T. pallidum*.

### Verification of Tp40 as an *in vivo*-induced antigen in *T. pallidum*

#### Verification of Tp40 as an *in vivo*-induced antigen in *T. pallidum* by monitoring the transcriptional levels in infected New Zealand rabbits

**Fig 3:**
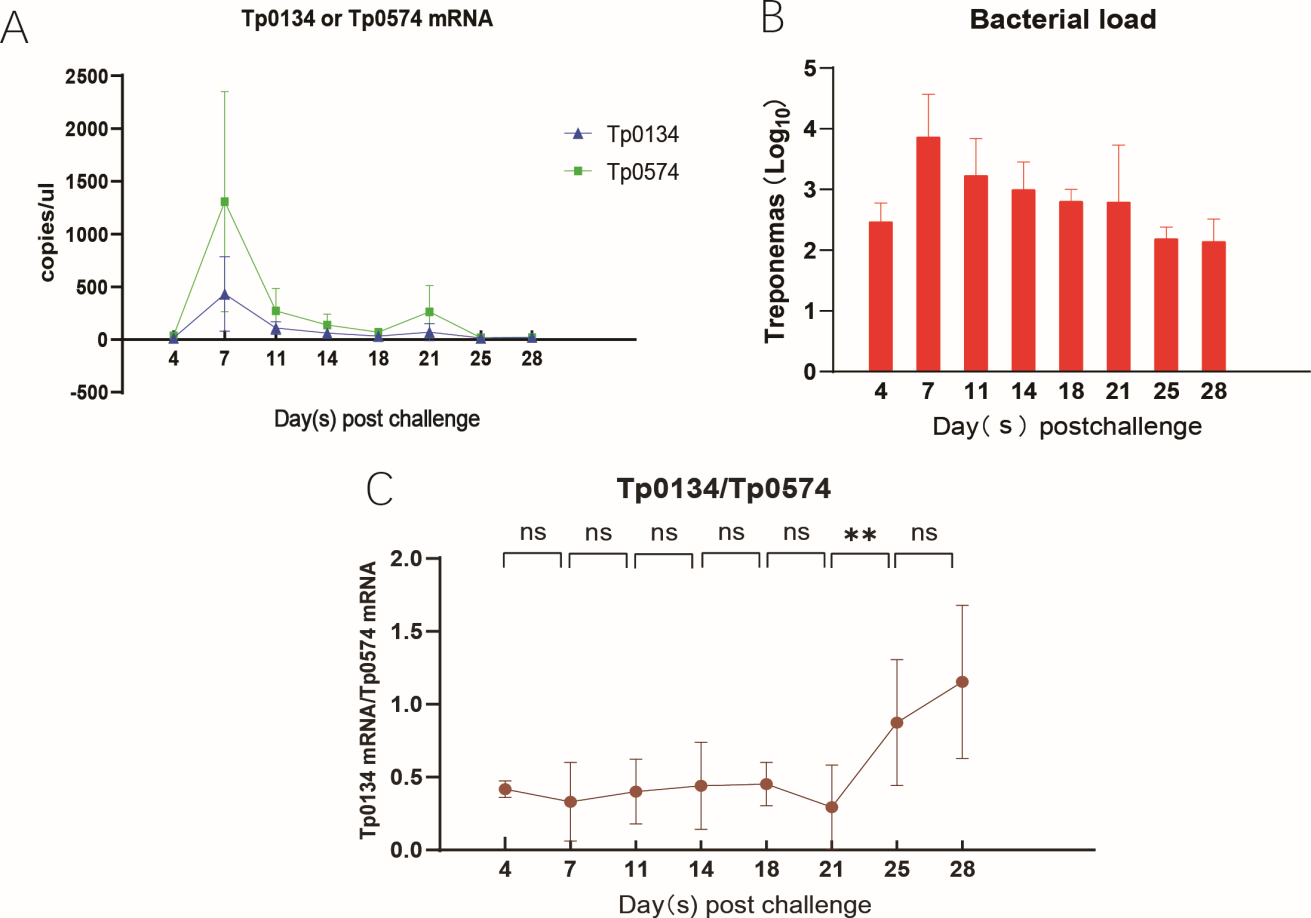
The mRNA results of Tp40/Tp0574 (**P < 0.01). (A) For message quantification, a biopsy from the leading edge of a dermal lesion was obtained from each of the four infected rabbits twice a week for 28 days. Each sample was amplified in triplicate. The data were reported as the mean values ± standard error (SE) for triplicate experiments. Vertical axis shows absolute quantification data for the Tp40 or Tp0574 message (blue or green bars), reflecting the absolute *T. pallidum* burden. (B) Changes in the load of *Treponema pallidum* in rabbit skin lesions. (C) Vertical axis shows real-time qPCR analysis of the Tp40 message normalized to Tp0547 mRNA (brown line) during the progression of primary syphilitic lesions in the rabbit model. Newman-Keuls Multiple Comparison Test was used to assess significant differences in Tp40 message level between time points whenever a significant difference between sample means was found by ANOVA. **P < 0.01; ns, no statistical significance.

Four syphilitic rabbit models were used, and RNA was extracted and reverse-transcribed from the skin lesions. RT-qPCR was performed to dynamically measure the mRNA levels of Tp40 and Tp0574. The normalized mRNA expression levels of Tp40 were obtained (F = 3.801, *P* < 0.05, Table 1), with each sample tested three times. The results are shown in Fig. 3.

**TABLE 1:** Τp0134/Tp0574 mRNA statistical results

Days of infection	Sample number	x ± s
4	4	0.37 ± 0.10
7	4	0.43 ± 0.30
11	4	0.31 ± 0.26
14	4	0.34 ± 0.32
18	4	0.41 ± 0.15
21	4	0.26 ± 0.31
25	4	0.87 ± 0.43
28	4	1.15 ± 0.53

From Fig. 3, it can be observed that the transcriptional level of Tp40 mRNA in the infected rabbit skin lesions remains stable during the early stages of infection (days 4–21), and then gradually increases (days 21–28). The transcriptional levels of Tp40 mRNA at days 25 and 28 are statistically significant compared to earlier time points (days 4–21) (*P* < 0.05), but there is no statistical difference between the two (*P* > 0.05). Tp0574 (Tp47) is a stable protein expressed by *T. pallidum*, and its mRNA level is positively correlated with the total amount of *T. pallidum* in the skin lesions. Therefore, we can observe a sharp decrease in the total amount of *T. pallidum* on day 11 (Fig. 3B), while the transcriptional level of Tp40 mRNA/Tp0574 mRNA shows an increasing trend on day 25, which is consistent with the *in vivo*-induced antigenic property of *T. pallidum* and suggests that Tp40 may play an important role during the latent stage of syphilis.

#### Verification of Tp40 as an *in vivo*-induced antigen in *T. pallidum* by monitoring the antibody levels in rabbits following infection with Live/Inactivated *T. pallidum*

**Fig 4:**
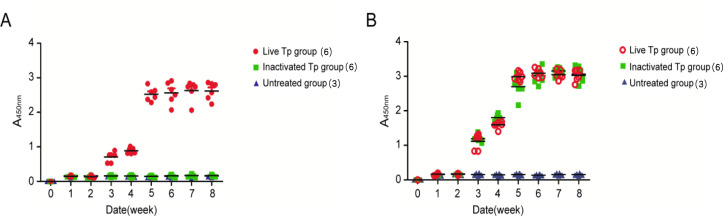
The changes of specific antibody levels of Tp40 and Tp92 in three groups at 1–8 weeks after infection The analysis of variance of repeated measurements showed that (**A**) the level of Tp40 specific antibody in active Tp group was significantly higher than that in Inactivated Tp group and Untreated group (*P* < 0.05), but (**B**) there was no significant difference in Tp92 specific antibody level between Live Tp group and Inactivated Tp group (*P* > 0.05).

In the group of rabbits infected with live *T. pallidum*, the testes of the New Zealand rabbits showed obvious redness and swelling around the second week after infection, and both the TPPA and RPR tests were positive, indicating successful infection. To determine if Tp40 is an *in vivo*-induced antigen of *T. pallidum*, an indirect ELISA was performed to compare the levels of specific antibodies against Tp40 and the control outer membrane protein Tp92 in rabbit sera collected at different time points. The results are shown in Fig. 4A. Compared to the groups infected with Inactivated *T. pallidum* and the control group, the group infected with live *T. pallidum* showed a significant increase in specific antibodies against Tp40 at each time point from weeks 3 to 8 (F values were 113.6, 454.2, 519.0, 527.2, 988.1, and 1,707.1, *P* < 0.05). The specific antibody levels against Tp92 in the group infected with live *T. pallidum* (Fig. 4B) showed an upward trend starting from week 2 and remained stable from weeks 3 to 5, with no statistically significant difference between the two groups at each time point from weeks 3 to 8 (F values were 0.99, 1.52, 2.86, 2.87, 2.99, and 2.91, *P* > 0.05). The trend of antibody changes indirectly indicates that the Tp40 protein exhibits the characteristics of an *in vivo*-induced antigen, while the outer membrane protein Tp92 does not possess this characteristic.

### Tp40 may provide potential diagnostic value for the course of syphilis

In previous studies, our research team validated the potential diagnostic value of *in vivo*-induced antigen proteins Tp0971, Tp0462, and Tp0768 for syphilis (14).

**Fig 5:**
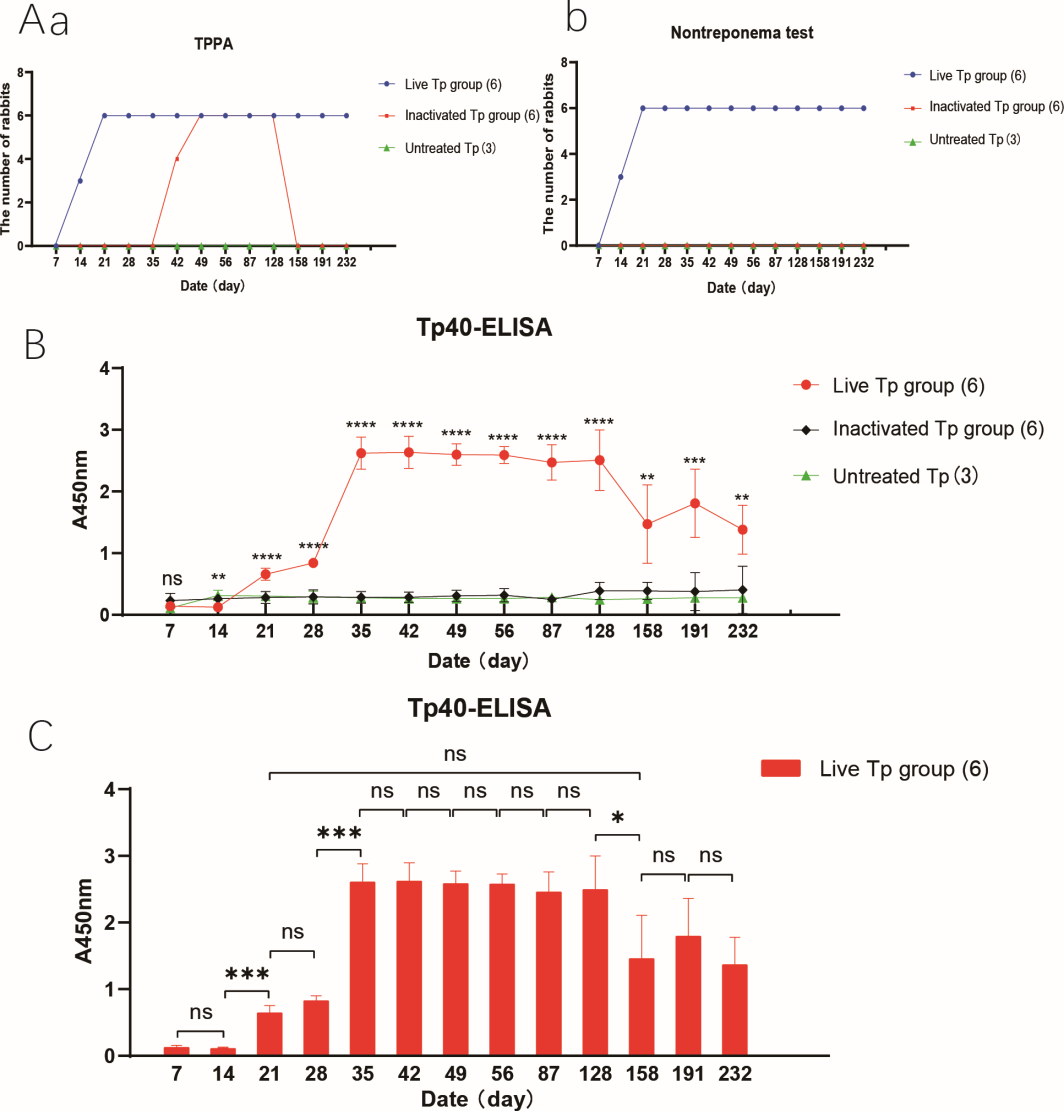
The results of animal serum detection at different time points. (A) Figure (a) displays the time to seropositive conversion and changes in the number of animals for the qualitative TPPA test in serum across the Live Tp group, Inactivated Tp group, and Untreated control group. Figure (b) illustrates the corresponding data for the qualitative non-treponemal test in serum, showcasing the time to seropositive conversion and variations in animal count within the same groups: Live Tp, Inactivated Tp, and Untreated control; (B) Active Tp group, Inactivated Tp group, and Untreated group Tp40-ELISA in A_450 nm_ OD value line chart; the "*" indicates statistical differences between the Live and Inactivated Tp groups and between the Live and Untreated groups, with no difference between the Inactivated and Untreated groups at all time points and therefore not marked in the figure. (C) Live Tp group showed a histogram of A_450 nm_ OD value. The data were analyzed by IBM SPSS Statistics 23 software. *, *P* < 0.05; **, *P* < 0.01; ***, *P* < 0.001; ****, *P* < 0.0001; ns, no statistical significance.

As shown in Fig. 5A, in the Live Tp group, three out of six New Zealand rabbits tested positive for TPPA and TRUST on day 14 after infection, and all six rabbits tested positive on day 21. The Tp40-ELISA and RPR results in the Inactivated Tp group and the Untreated group were consistently negative. Interestingly, the Inactivated Tp group showed a positive TPPA result at day 42, but negative results at day 158 and onwards. Since the bacteria used for the Inactivated Tp group were treated to be non-viable, the positive result may be due to inherent antigen stimulation of the inactivated bacteria, which triggers the production of *T. pallidum* antibodies by the animal’s immune system. The subsequent negative results may be attributed to the degradation of the inactivated bacterial antigens in the body, which weakly stimulates the immune memory of the host, leading to the gradual disappearance of the induced antibodies. This indirectly suggests that live *T. pallidum* may persist in the Live Tp group. Furthermore, the level of Tp40 antibody in Live Tp group was significantly different from that in Inactivated Tp group and an Untreated group from day 14 to day 232 after inoculation (**, *P* < 0.01; ***, *P* < 0.001; ****P < 0.0001, Fig. 5B). And it increased at day 21 day (*, *P* < 0.05, Fig. 5C), and then decreased gradually after maintaining a stable high level from day 35 to day 128 (ns, *P* > 0.05, Fig. 5C). The specific antibody levels against Tp40 at days 158, 191, and 232 reached no statistical significance compared to the results at days 21 and 28 (ns, *P* > 0.05, Fig. 5C). In the later stages of infection, significant changes in antibody levels indicate that the bacterial load and protein expression frequency of the *T. pallidum* may be reduced by the host’s immune response, or both may occur. The dynamic changes in antibody levels detected by Tp40-ELISA, compared to the qualitative results of TPPA and non-treponema test, more accurately reflect the activity changes of *T. pallidum* in the host, indicating the potential diagnostic value for *T. pallidum* invasion or load changes.

### Tp40-ELISA shows high consistency with TPPA, LZ syphilis screening ELISA, and RPR tests, indicating high diagnostic value

To investigate the potential diagnostic value of Tp40 in clinical diagnosis, we collected 468 clinical serum samples, as described earlier. The diagnostic positivity rates of Tp40-ELISA for primary, secondary, tertiary, latent, and congenital syphilis were 96.67% (58/60), 97.5% (39/40), 100% (60/60), 92.5% (37/40), and 100% (14/14), respectively. Among them, there were six cases of clinical diagnosis with negative Tp40-ELISA results (false negatives) (Table 2).

**TABLE 2:** The positive rate of Tp40 in the diagnosis of syphilis in different clinical stages

Syphilis stage	Positive samples	Tp40-ELISA negative			Tp40-ELISA positive
		Number	Age	The titers of RPR	Cases (positive rate%)
Primary syphilis	60	2	42–76	Negative ~1∶4	58 (96.67%)
Secondary syphilis	40	1	62	Negative ~1∶4	39 (97.5%)
Tertiary syphilis	60	0	–^*a*^	–	60 (100%)
Latent syphilis	40	3	44–74	Negative ~1∶1	37 (92.5%)
Fetal syphilis	14	0	–	–	14 (100%)

^*a*^ “–” indicates that the information is unavailable.

The results of Tp40-ELISA were compared with LZ-ELISA and Shanghai Kehua RPR test for the 468 serum samples (Table 3), with concordance rates of 95.30% and 89.10%, and Kappa values of 0.906 and 0.779, respectively. Both values were greater than 0.75, indicating a high consistency between Tp40-ELISA and these two methods, with the consistency between Tp40-ELISA and LZ-ELISA being higher than that with RPR.

**TABLE 3:** Comparison of diagnostic evaluation between Tp40-ELISA and TPPA, Lizhu syphilis screening ELISA kit and RPR kit

Diagnostic test	Tp40-ELISA		Sensitivity (%)	Specificity (%)	Coincidence rate (%)	Kappa value
	+	−				
TPPA (+)	208	6	97.19 (97.20)	96.85 (96.30)	97.01	0.94
TPPA (−)	8	246				
LZ-ELISA (+)	208	20	91.23 (92.25)	99.17 (99.05)	95.30	0.906
LZ-ELISA (−)	2	238				
RPR (+)	181	26	87.44 (90.08)	90.42 (87.86)	89.10	0.779
RPR (−)	25	236				

TPPA is currently recognized as a serological diagnostic test for syphilis with good sensitivity and specificity. Using TPPA as the gold standard, the sensitivity of Tp40-ELISA in detecting the 468 serum samples was 97.20%, and the specificity was 96.85%. The positive predictive value and negative predictive value were 96.30% and 97.20%, respectively, and the overall agreement rate was 97.01% (Table 3). These preliminary results suggest that Tp40-ELISA performs well in terms of diagnostic efficacy.

## DISCUSSION

*In vivo*-induced antigen refers to the neoantigen produced by the host cell after the infection of bacteria, viruses, and other pathogens. These antigens can be proteins encoded by viral or bacterial genes, or they can be some abnormal proteins produced by host cells in response to the stimulation of pathogen infection. Induced antigens produced by pathogens (such as *Mycobacterium tuberculosis*) may play a role in immune evasion, invasion and diffusion, growth, and proliferation during human infection, so they reflect the activity and content of pathogens to a certain extent (24). There are still many gaps in the research on the hypothetical protein Tp40, especially regarding its similarity to other pathogen-induced antigens. In this study, we used bioinformatics tools to predict the signal peptide cleavage sites and transmembrane sequences of the protein. Based on this, we hypothesized that Tp40, which consists of 376 amino acids, is a non-transmembrane-secreted protein with a signal peptide sequence. To further validate this hypothesis, we conducted a membrane localization analysis of the protein.

The membrane localization study of Tp40 was conducted using an immunofluorescence analysis with the antiserum generated from recombinant *T. pallidum* lipoprotein. The key technique used in this study was the gel microdroplet method, which maintained the integrity of the *T. pallidum* outer membrane throughout the localization experiment (25). The complete details of the technique process have been published by Luthra et al. (19). This method has been successfully applied to localize *T. pallidum* proteins such as Tp92 (Tp0326), Tp47 (Tp0574), and Tp0136 (26). We used *T. pallidum* flagellar sheath protein FlaA2 as a control protein for the periplasmic space (23). The preliminary experimental results indicated that the Tp40 hypothetical protein is located in the periplasmic space outside the inner membrane.

The localization of Tp40 in the periplasmic space and its non-transmembrane nature further suggests that this protein may be a non-membrane-secreted protein, which is consistent with the characteristics of “non-cellular intrinsic membrane proteins” induced by antigens in *T. pallidum*. To further validate whether Tp40 is an *in vivo*-induced antigen, we conducted *in vivo* antigenicity identification by analyzing the transcription levels of Tp40 and the corresponding specific antibody levels in New Zealand rabbits infected with *T. pallidum*. The experimental study on the transcription of *T. pallidum* genes in the early stages of *T. pallidum* infection was initially reported by Ke Wujian et al. in their study on the mRNA expression of Tp0136 protein in a rabbit infection model through intradermal inoculation of *T. pallidum* (21). This experimental method provided insights into the transcription of *T. pallidum* genes in the host during the early stages of infection for a certain period. Tp47 is a membrane protein of *Treponema pallidum*. Due to its high content, stable expression, and strong immunogenicity in *Treponema pallidum*, Tp47 has been widely used in laboratory antibody detection and is often used in the study of pathogenic mechanisms. In addition, Tp47 coding gene Tp0574 is often selected as the target gene for laboratory gene detection and used to measure bacterial load (21, 27–29). Therefore, it is recognized as a stable bacterial antigen. In the experimental process, we chose to detect the mRNA level of Tp0574 to reflect the total amount of *T. pallidum* (30). When *T. pallidum* infects an animal, it migrates rapidly and invades the tissues and organs of the host, so the *T. pallidum* in the skin lesions gradually decreases. The dynamic changes in Tp0574 mRNA levels reflected a sharp decrease in *T. pallidum* load at the site of skin lesions on the 11th day after rabbit infection, while the transcription level of Tp40/Tp0574 showed an upward trend in the later stage of infection (21st day). In addition, with the same load, the ratio of Tp40/Tp0574 on days 25 and 28 was significantly higher than that on day 4, indicating that the expression of Tp40 was increasing, indicating that it may play a potential role in the evasion of immunity, proliferation, invasion, and diffusion of *T. pallidum* in the host.

Although Tp40 is not directly located on the outer membrane of *T. pallidum*, its expression is increased when *T. pallidum* is active in the host, while the expression of inactivated *T. pallidum* is not increased, suggesting that it reflects the content and activity of the *T. pallidum* to a certain extent. The Tp40 protein located in the periplasmic space between the inner and outer membranes can still cause an immune response in the host, which may be related to its antigen-inducing properties *in vivo*. During the infection of *T. pallidum*, this protein may be expressed and secreted into the outside world or transferred to the outer membrane to play a certain function and cause an immune response. Furthermore, after the antigen-presenting cells phagocytize spirochaeta, the bacterial structure is destroyed, so that Tp40 will also be exposed around the cytoplasm, and then induce antibody production. We validated whether Tp40 is an *in vivo*-induced antigen by measuring the levels of specific antibodies against Tp40 in animal sera. Tp92 is a well-known non-*in vivo*-induced intrinsic outer membrane protein (31), and we used it as a control protein. The experimental results showed a significant increase in specific antibody levels against Tp40 in the sera of rabbits in the Live Tp group, while the levels remained unchanged in the Inactivated Tp group and the blank control group. On the other hand, specific antibody levels against Tp92 increased in both the Live Tp group and the Inactivated Tp group, but not in the blank control group. This result indicates that Tp40 shows a specific expression pattern, which is highly expressed when *T. pallidum* is in an active stage of infection within the host. It suggests that Tp40 might be involved in the proliferation process, invasive behavior, and spreading activities of *T. pallidum* within the host. This is consistent with the typical characteristics of *in vivo*-induced antigens and conforms to the expected role it plays in the dynamic process of infection. Combining the results of the protein expression level changes mentioned above, we can preliminarily infer that Tp40 is an *in vivo*-induced antigen of *T. pallidum*.

Currently, the diagnosis of syphilis in clinical practice mainly relies on serological tests, which ideally should be inexpensive, easy to perform, highly specific, and sensitive. Previous studies have found that *in vivo*-induced antigens such as Tp0462 can be used as novel serological diagnostic antigens for syphilis (14). Therefore, we conducted a preliminary analysis and evaluation of whether Tp40 is suitable as another novel candidate antigen for syphilis diagnosis.

As it was not possible to obtain human sera immunized with inactivated *T. pallidum* vaccine, we established a New Zealand rabbit infection model. Clinical test kits TPPA, TRUST, or RPR, and the constructed Tp40 indirect ELISA (Tp40-ELISA) were used to detect rabbit sera collected at different time points in each experimental group. During the experiment, we found that the TPPA test results for the sera of the Inactivated Tp group were positive on day 42 after inoculation, but turned negative on day 158. From an immunological perspective, this can be explained by the gradual decrease in the amount of inactivated *T. pallidum* in the host compared to the Live Tp group, leading to a gradual decline in memory B cells secreting *T. pallidum* antibodies (32). Due to the immune resistance of New Zealand rabbits to *T. pallidum*, the spirochete may exhibit fluctuating patterns of activity and latency within the host. In the Live Tp group, the Tp40-ELISA began to decline after 128 days, potentially reflecting a reduction in spirochete load or the frequency of protein expression, or both. This suggests that Tp40-ELISA may be more indicative of changes in Tp activity within the host compared to the TPPA or non-treponemal tests, and holds promise for use in treatment efficacy evaluation.

We further investigated the clinical diagnostic value of Tp40-ELISA by testing 468 clinical serum samples. The results showed that Tp40-ELISA exhibited higher consistency in the diagnosis of late-stage and congenital syphilis screening (Table 2). Among the six serum samples that tested negative for Tp40-ELISA but positive for RPR, all were from patients over 40 years old, and the RPR titers were all less than 1:4. We speculate that the low consistency between Tp40-ELISA and RPR in these cases may be due to the influence of factors such as advanced age, low immune levels, and serum resistance (serofast), which may indicate that these patients have undergone a process of syphilis infection and subsequent cure, leading to the conversion from positive to negative in Tp40-ELISA results. Meanwhile, compared to other diagnostic kits such as LZ-ELISA and Shanghai Kehua RPR kit (Table 3), Tp40-ELISA demonstrated a high level of consistency, with a kappa value of 0.906 for LZ-ELISA and 0.779 for RPR. Using the widely accepted TPPA as the gold standard for sensitivity and specificity, Tp40-ELISA showed a sensitivity of 97.20%, specificity of 96.85%, positive predictive value of 96.30%, negative predictive value of 97.20%, and an overall agreement rate of 97.01% (Table 3), which indicates a good clinical diagnostic value for Tp40-ELISA in syphilis. Despite its high sensitivity and specificity, making it well-suited for serological diagnosis of syphilis, the TPPA employs whole-treponema antigen-sensitized gelatin particles, which involves a cumbersome preparation and labeling process and results in higher production costs. Furthermore, TPPA cannot be used to assess treatment efficacy or active infection. In contrast, the ELISA method, utilizing a single antigen with equally high sensitivity and specificity, offers ease of operation and lower costs, making it more practical for primary healthcare units. Additionally, certain antigens used in ELISA may have broader application prospects, potentially providing more effective references in evaluating treatment efficacy, recurrent infections, or treatment failures. Therefore, Tp40 may be the antigen that monitors the activity of treponema pallidum in the host, and the development of an ELISA to detect antibodies induced by such antigens may have higher clinical significance.

The changes in the levels of specific antibodies produced by the host in response to Tp40-specific antigen stimulation in the animal experiments, the changes in Tp40 antibody levels seem to more accurately reflect the cure of syphilis in the host. Tp40-ELISA has the potential to become a new method for evaluating the effectiveness of clinical syphilis treatment or the progression of late-stage syphilis. However, due to the lack of animal and clinical trial verification before and after treatment, the conclusions supported by our results are still very weak. Therefore, it is necessary to validate the efficacy assessment of the Tp40-ELISA method in both animal experiments and clinical trials, with comparative analysis conducted using quantitative non-treponemal tests and the TPPA method. We are currently conducting a multicenter collection of serum samples from patients before and after treatment, accompanied by animal experimental research. Our aim is to explore the potential use of infection-dependent antigens, including Tp40, for assessing the treatment efficacy of syphilis treatment. Additionally, in future research, we will attempt to combine the advantages of the previously optimized *T. pallidum* membrane lipoprotein and infection-dependent antigen epitopes to construct *T. pallidum* multi-antigen multi-peptide structures, aiming to achieve more ideal results in syphilis clinical diagnosis and prognosis assessment.

### Conclusion

This study is the first to investigate the protein Tp40 encoded by the Tp0134 gene, which is a potential *in vivo*-induced antigen located in the intimal periplasm. Tp40 can serve as a novel syphilis diagnostic candidate antigen for serological diagnosis and evaluation of treatment efficacy or late-stage syphilis. This study provides a valuable reference for further exploring the functional mechanism of the Tp40 protein and its important significance in clinical diagnosis.

## SUPPLEMENTAL MATERIAL

Supplemental material spectrum.02799-24-s0001.docx Fig. S1 to S3.

ASM does not own the copyrights to Supplemental Material that may be linked to, or accessed through, an article. The authors have granted ASM a non-exclusive, world-wide license to publish the Supplemental Material files. Please contact the corresponding author directly for reuse.
