# Supplementary material for: Tp40: a new potential prognostic and diagnostic marker for syphilis
Source: Microbiol Spectr. 2025 Feb 11;13(3):e02799-24. doi: 10.1128/spectrum.02799-24 (PMC11878081; doi:10.1128/spectrum.02799-24)
Supplement: Supplemental material — Fig. S1 to S3. [file spectrum.02799-24-s0001.docx]

Supplementary Materials for

**Tp40: A New Potential Prognostic and Diagnostic Marker for Syphilis**

Jiangchen Yao *et al.*

*Corresponding author: Zhao Feijun. Email: nhdxzhfj@163.com

**This PDF file includes:**

Fig. S1

Fig. S2

Fig. S3


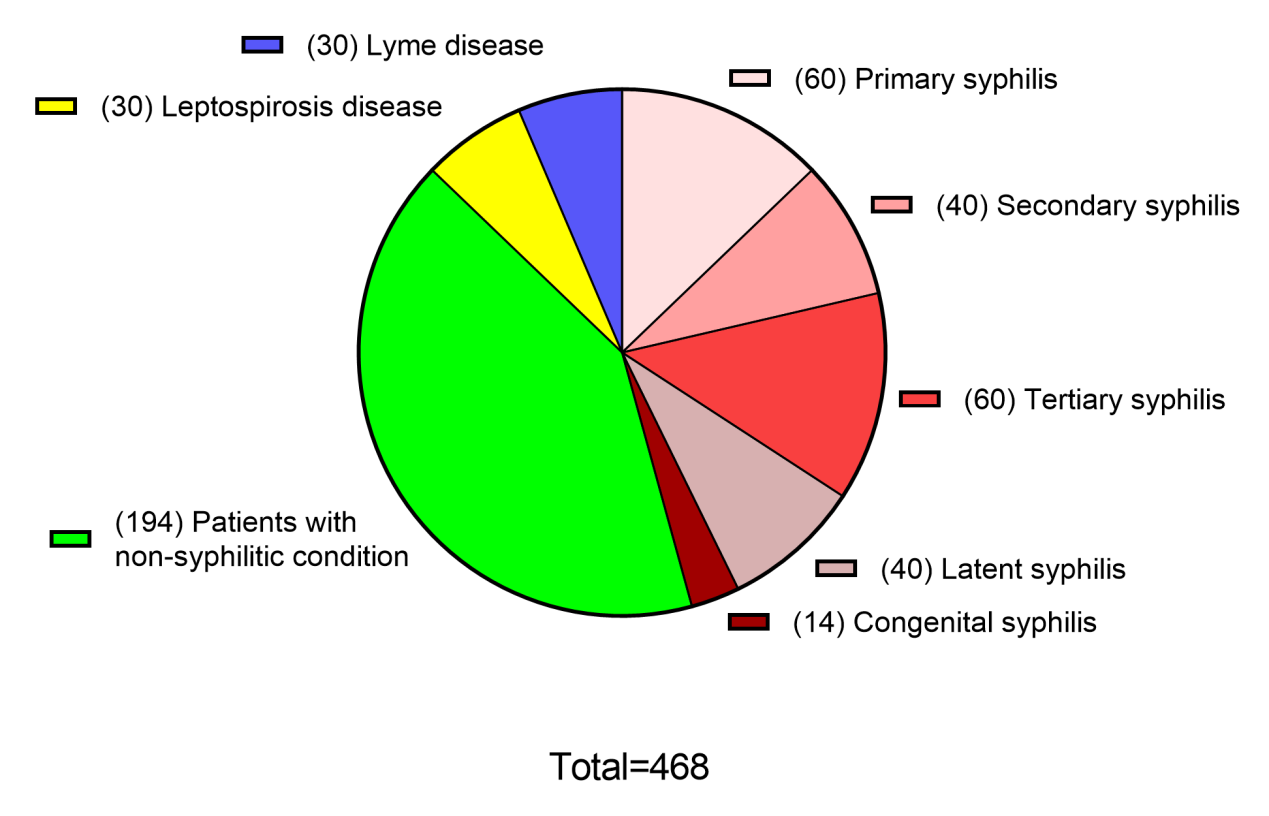


**Fig S1.The pie chart of 468 clinical serum samples of syphilis detecation.** The number of syphilis samples in brackets.


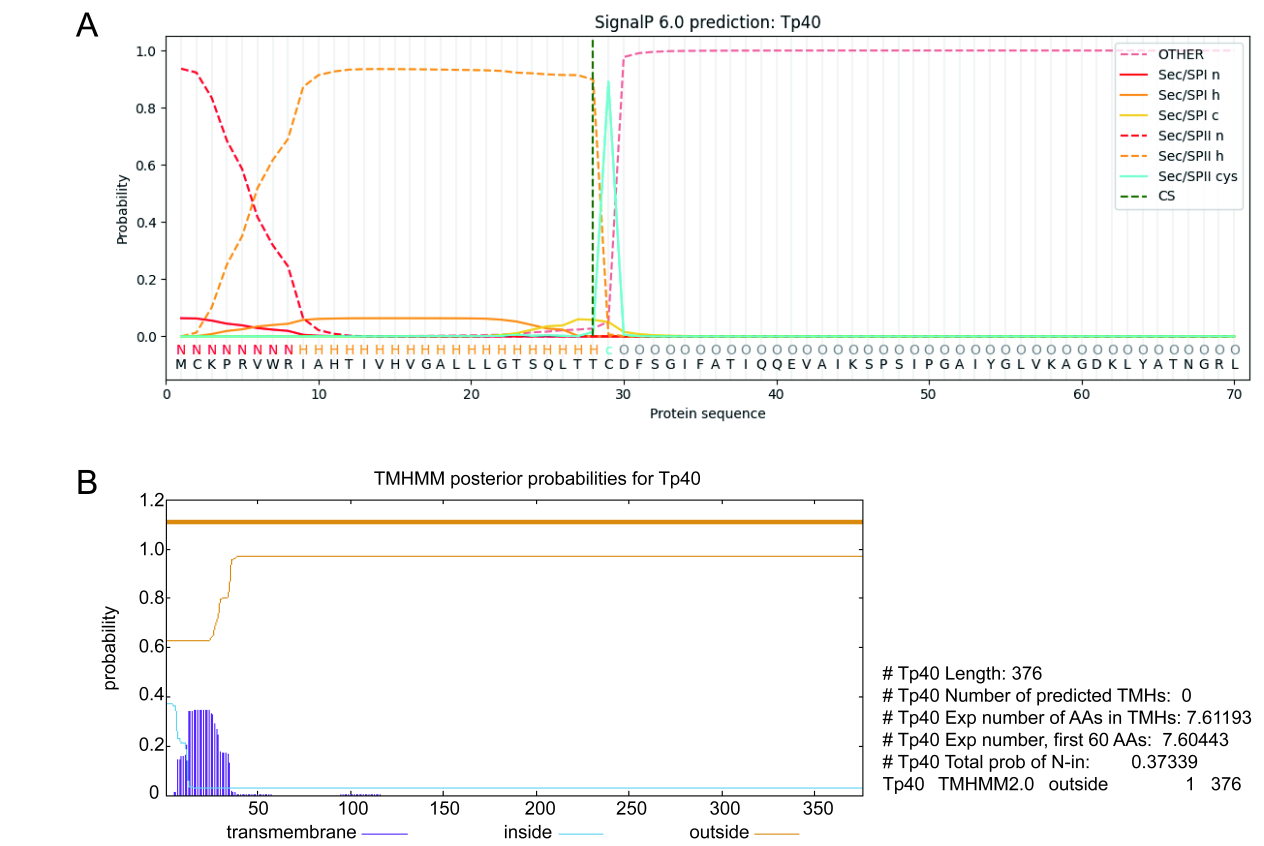


**Fig S2.The predictions of signal peptide and transmembrane helices of Tp40.**

**(A)Signal peptides and cleavage sites of Tp40 by SignalP 6.0.** The signal peptide prediction is consistent with the database annotation.Tp40 Prediction: Lipoprotein signal peptide (Sec/SPII); Cleavage site between pos. 29 and 30; Probability 0.9991. Sec/SPI: "standard" secretory signal peptides transported by the Sec translocon and cleaved by Signal Peptidase I (Lep)；Sec/SPII: lipoprotein signal peptides transported by the Sec translocon and cleaved by Signal Peptidase II (Lsp)；Tat/SPI: Tat signal peptides transported by the Tat translocon and cleaved by Signal Peptidase I (Lep)；Tat/SPII: Tat lipoprotein signal peptides transported by the Tat translocon and cleaved by Signal Peptidase II (Lsp)；Other: No signal peptide at all.n-region: The n-terminal region of the signal peptide, reported for Sec/SPI, Sec/SPII, Tat/SPI and Tat/SPII. Cysteine(CS): The conserved cysteine in +1 of the cleavage site of Lipoproteins that is used for Lipidation.

**(B)Graphical illustration of transmembrane domain analysis of Tp40 by TMHMM-2.0.** Number of predicted TMHs: The number of predicted transmembrane helices; Exp number of AAs in TMHs: The expected number of amino acids in transmembrane helices. If this number is larger than 18 it is very likely to be a transmembrane protein (OR have a signal peptide); Exp number, first 60 AAs: The expected number of amino acids in transmembrane helices in the first 60 amino acids of the protein. If this number more than a few, you should be warned that a predicted transmembrane helix in the N-term could be a signal peptide; Total prob of N-in: The total probability that the N-term is on the cytoplasmic side of the membrane; Possible N-term signal sequence: a warning that is produced when “Exp number, first 60 AAs” is larger than 10.

**
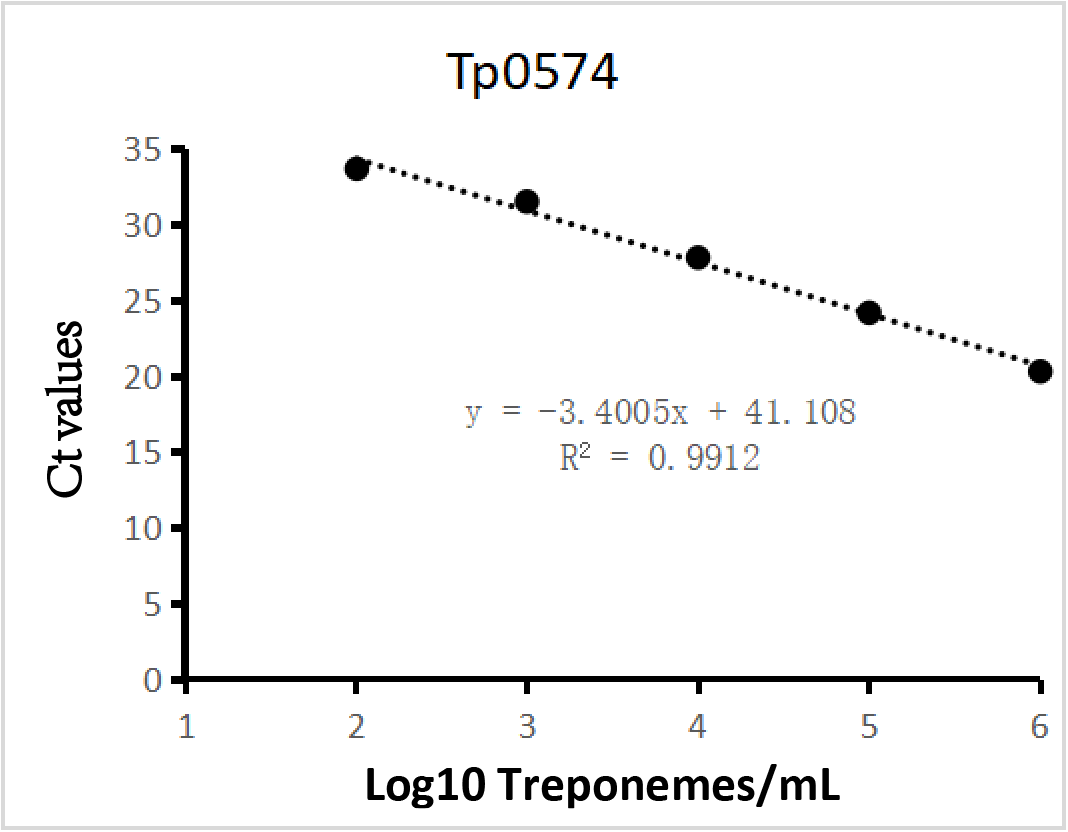
**

**Fig S3. Standard curve for qPCR amplification of Tp0574 mRNA.**

A suspension of T. pallidum was prepared at a concentration of 1×10^7^ organisms/mL. When the gradient-diluted T. pallidum-mRNA was detected using the Tp0574 RT-qPCR (Reverse transcriptional quantitative PCR) methods, the Ct values increased in a gradient manner. Linear regression analysis revealed slopes of -3.4005, with R² values exceeding 0.995. The result demonstrates that RT-qPCR methods exhibit excellent linearity and amplification efficiency in detecting T. pallidum-mRNA.
